# Personalized connectivity-based network targeting model of transcranial magnetic stimulation for treatment of psychiatric disorders: computational feasibility and reproducibility

**DOI:** 10.3389/fpsyt.2024.1341908

**Published:** 2024-02-14

**Authors:** Zhengcao Cao, Xiang Xiao, Cong Xie, Lijiang Wei, Yihong Yang, Chaozhe Zhu

**Affiliations:** ^1^ State Key Laboratory of Cognitive Neuroscience and Learning, Beijing Normal University, Beijing, China; ^2^ School of Arts and Communication, Beijing Normal University, Beijing, China; ^3^ Neuroimaging Research Branch, National Institute on Drug Abuse, National Institutes of Health, Baltimore, MD, United States; ^4^ IDG/McGovern Institute for Brain Research, Beijing Normal University, Beijing, China; ^5^ Center for Collaboration and Innovation in Brain and Learning Sciences, Beijing Normal University, Beijing, China

**Keywords:** transcranial magnetic stimulation, psychiatric disorders, personalized targeting, individualized functional connectivity, stability of functional connectivity

## Abstract

Repetitive transcranial magnetic stimulation (rTMS) holds promise for treating psychiatric disorders; however, the variability in treatment efficacy among individuals underscores the need for further improvement. Growing evidence has shown that TMS induces a broad network modulatory effect, and its effectiveness may rely on accurate modulation of the pathological network specific to each disorder. Therefore, determining the optimal TMS coil setting that will engage the functional pathway delivering the stimulation is crucial. Compared to group-averaged functional connectivity (FC), individual FC provides specific information about a person’s brain functional architecture, offering the potential for more accurate network targeting for personalized TMS. However, the low signal-to-noise ratio (SNR) of FC poses a challenge when utilizing individual resting-state FC. To overcome this challenge, the proposed solutions include increasing the scan duration and employing the cluster method to enhance the stability of FC. This study aimed to evaluate the stability of a personalized FC-based network targeting model in individuals with major depressive disorder or schizophrenia with auditory verbal hallucinations. Using resting-state functional magnetic resonance imaging data from the Human Connectome Project, we assessed the model’s stability. We employed longer scan durations and cluster methodologies to improve the precision in identifying optimal individual sites. Our findings demonstrate that a scan duration of 28 minutes and the utilization of the cluster method achieved stable identification of individual sites, as evidenced by the intraindividual distance falling below the ~1cm spatial resolution of TMS. The current model provides a feasible approach to obtaining stable personalized TMS targets from the scalp, offering a more accurate method of TMS targeting in clinical applications.

## Highlights

- Replaced the group-averaged functional connectivity with individualized functional connectivity in the network targeting model, offering the potential for higher accurate network targeting in personalized TMS.
- Demonstrated a significant variability in optimal individual stimulation sites with the Human Connectome Project dataset, underscoring the necessity for further improvements in personalized approaches.
- Employed approaches such as extended resting-state functional MRI scans and a spatial cluster method to enhance TMS targeting stability, ensuring the optimal TMS target site aligns with the spatial resolution of TMS.

## 1 Introduction

Transcranial magnetic stimulation (TMS) is a non-invasive neuromodulation technology with ~1cm spatial resolution (1, 2). TMS has received FDA approval as a safe and effective therapy for patients with major depressive disorder (MDD) who do not respond to behavioral or pharmacological treatment and has also proved its potential as a novel treatment for other psychiatric disorders, including schizophrenia (3, 4). Though the general efficacy is demonstrated for the TMS-based treatment, its clinical utility is limited by the heterogeneous outcomes in individual patients, even when their clinical conditions are similar.

Differences in the morphology and functional connectivity (FC) of individual brains may account for the heterogeneous outcomes of TMS (5–7). Traditionally, the TMS coil is set according to scalp landmarks, e.g., EEG position F3, anterior 5-cm from the motor evoked potential (MEP) hop-spot for MDD or the mid-point of T3 and P3 for schizophrenia with auditory verbal hallucinations (AVH)(4). Such landmark-based targeting strategies and even more advanced neuronavigation techniques may oversimplify the physiological process of how TMS generates the modulation effect on the human brain system. First, the E-filed distribution highly depends on the intracranial geometry of the human brain (8). Thus, even when the TMS coil is set in an identical spot on the patient’s scalp, the actual excited cortical area can vary significantly among different subjects or even on the same subject but with varied coil orientations (9). Second, the associative cortical areas that are commonly targeted for treating psychiatric disorders, e.g., the dorsal lateral prefrontal cortex (DLPFC) for MDD and temporoparietal junction (TPJ) for schizophrenia with AVH, exhibit the highest levels of interindividual variation in terms of structural morphology, neuronal function, and connection (10–17). As a result, varied networks can be engaged in the effect field of the TMS stimulation through mono-/multi- synaptic connections to the brain areas that directly receive TMS stimulations, which is considered to account for the heterogeneous treatment efficacies of TMS.

Based on the observation that the treatment efficacy is associated with the extent to which the pathological network of a given disorder is engaged in the stimulation network, our previous work proposed a network targeting accuracy (NTA) model for guiding TMS coil placement for individual patients (18). Considering the reliability of the targeting result, the NTA model was initially based on group-averaged functional connectivity.

Individual functional connectivity has several advantages over normative or average connectomes (19). First, a study compared group-based targeting with individualized targeting in TMS and found that individualized stimulation sites improved the reliability of TMS-evoked responses, particularly in highly variable task-positive networks, such as the dorsal attention network (DAN)(20). Second, studies comparing individual and normative connectomes have shown similar results in predicting clinical responses, but a trend toward better prediction was observed with individual data (6, 13, 21, 22).

A major challenge for incorporating individual FC into the NTA model is the relatively low signal-to-noise ratio (SNR) of resting-state functional magnetic resonance imaging (rsfMRI). The low SNR of rsfMRI in FC calculations can lead to inaccurate measurements of correlation values, as the weak brain signal compared to noise may overshadow the actual underlying FC patterns (23–25). For this reason, it makes FC-based approaches unstable (26) and gives ambiguous guidance for setting the TMS coil (27).

Currently, strategies have been proposed to reduce the spurious FC variance introduced by the data acquisition. One strategy to enhance FC’s stability is to augment the number of data points or repetitions in rsfMRI. By extending the scan duration, a more comprehensive and stable evaluation of FC can be achieved, attributed to the reduction of noise (23), increased statistical power, and capture of the temporal dynamics of brain activity (24). Previous studies have demonstrated the beneficial impact of increased scan duration on the stability of individual FC (16, 28).

When FC is stable, it may indicate the presence of a robust pathway through which TMS exerts its effects on individuals. The establishment and stability of this pathway enable more accurate and effective targeting of specific brain regions, which in turn contributes to more favorable treatment outcomes. Another strategy category is to improve stability by spatially averaging the FC map, or the ‘cluster’ method, which calculates the center-of-gravity of the largest cluster (29, 30). In MDD, the cluster method has demonstrated its utility in reducing the within-subject instability while keeping the between-subject variance for the optimized treatment site (29).

In the present study, our objective was to evaluate the stability of a personalized FC-based network targeting model in individuals by targeting pathological networks of MDD or schizophrenia with AVH. We utilized two-day rsfMRI scans from the Human Connectome Project (HCP) dataset to assess the instability of the model, specifically focusing on the stimulation network, NTA map, and intraindividual distance. To address this stability challenge, we employed two strategies: longer scan time and the cluster method, aimed at improving the accuracy of identifying the optimal individual site. To ensure the generalizability of the model across different psychiatric disorders, we conducted stability validation in both MDD and schizophrenia with AVH.

## 2 Methods

### 2.1 Overview

**Figure 1:**
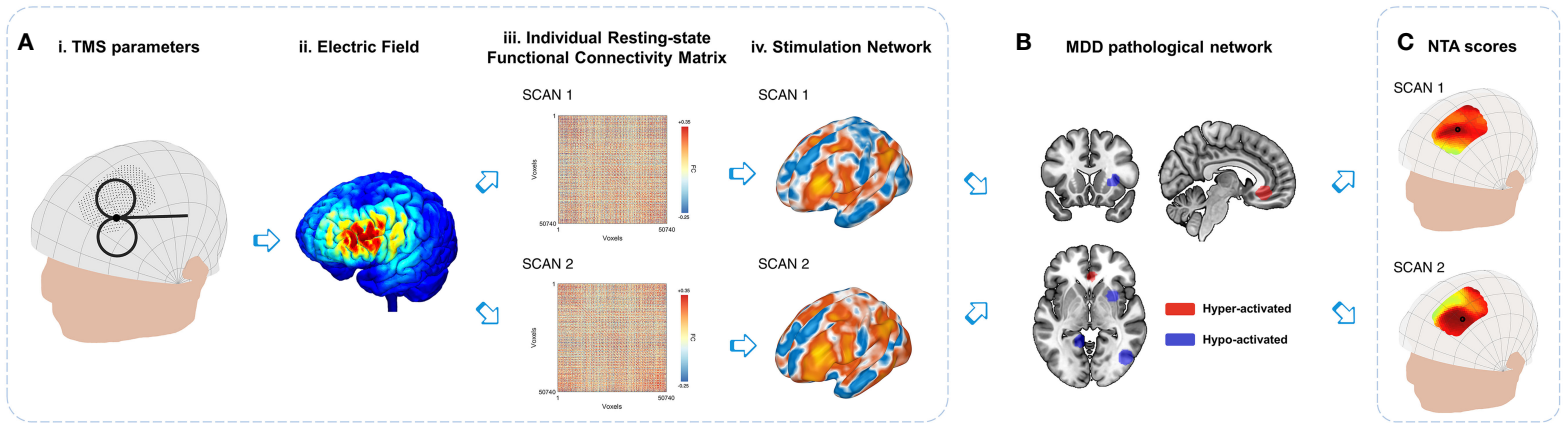
Variations in the personalized NTA model. **(A)** Stimulation network: TMS administered with specific combinations of parameters (i) from the search space results in an E-field (ii) that directly affects the local cortical region. The variances in individual resting-state functional connectivity from different scans (iii) contribute to the variations in the stimulation network (iv) within the stimulated cortical region. **(B)** Comparison with MDD pathological networks: The stimulation networks exhibiting spatial anti-correlation represent the MDD NTA score, which reflects the estimated efficacy of TMS (18). **(C)** For the entire search space, the NTA map is generated by considering all parameter combinations, with the optimal target site indicated by a black circle. The figure illustrates the variations in the NTA map and optimal target site.

This study aims to examine the variance of a personalized FC-based network targeting model using different rsfMRI scans and to reduce the variances through two strategies. Compared to a network targeting model based on group-averaged FC, the personalized FC-based network targeting model utilizing individual FC can better capture interindividual differences (**Supplementary Figure S1**). Regarding the variance of individual functional connectivity, there are two sources of variations. The first is the desirable variations, including inter-individual differences in network organization and connectivity strength (16, 28). The second is the undesirable variations, including unwanted technical effects or the influence of rsfMRI nuisance variables (23, 31, 32), which contribute to the variances observed in the stimulation network, NTA map, and optimal targets (**Figure 1**). For the current study, we aim to assess these technical variations and propose two strategies to mitigate them: extending the duration of rsfMRI scans to enhance the signal-to-noise ratio of individual rsfMRI data and employing alternative searching methods to identify optimal targets.

### 2.2 Participants

Two cohorts from the HCP-young adult dataset, namely the ‘100 unrelated subjects’ (27, 33) and ‘HCP Retest Data’ (34), were included in the current study. The resulting dataset consists of 134 participants [80 females, age 29.7 ± 3.5 years]. The rsfMRI data acquisition parameters in the database were TR=720 ms, TE=33 ms, flip angle=52°, FOV=208×180 mm², voxel size=2×2×2 mm³, and a multi-band factor of 8. The anatomical MRI volume size was 0.7×0.7×0.7 mm³. The anatomical MRI and rsfMRI data of the participants were used to construct E-field and FC separately.

Each participant underwent four fMRI scans on consecutive days. Two data acquisition sessions were conducted on each day, with each session comprising two 14-minute and 33-second runs (1200 volumes each) with right-to-left and left-to-right phase encodings. During scanning, participants were instructed to keep their eyes open and fixate on a projected bright cross-hair on a dark background.

### 2.3 rsfMRI data pre-processing

The rsfMRI data from the HCP dataset were preprocessed with the DPABI toolbox (35), which included the following steps: 1) elimination of the first ten time points; 2) correction for slice timing; 3) realignment of the functional image to correct for head motion; 4) regression of nuisance signals estimated from the signals of white matter, CSF and the mean global signal of gray matter(36); 5) 0.01~0.1Hz band-pass filtering; 6) The functional images were co-registered to scalp-extracted anatomical images and then normalized into MNI space (3 mm × 3 mm × 3 mm) with the DARTEL algorithm (37) and 7) spatial smoothing (kernel FWHM 6 mm × 6 mm × 6 mm).

### 2.4 Compute personalized NTA for MDD and schizophrenia with AVH

#### 2.4.1 Search space

In our study, for MDD, we utilized a cranial search space consisting of 462 scalp positions within a continuous proportional coordinate system (CPC) (38). This cranial search space covered a broad area of the left dorsolateral prefrontal cortex, defined by 20 mm radius spheres centered at BA9 [MNI -36, 39, 43], BA46 [MNI -44, 40, 29], Beam-F3 [MNI -37, 26, 49], and “5-cm” TMS site [MNI -41, 16, 54] (4, 29, 38). The coordinates in the CPC search space were *p_NZ_* ∈[0.15, 0.43] and *p_AL_* ∈[0.27, 0.43], as shown in **Supplementary Figure S2Ai**. Similarly, for the participants with schizophrenia AVH, we utilized a cranial search space consisting of 246 CPC positions. The search space covered a broad area of the left TPJ and left Wernicke’s area, defined by 20 mm radius spheres centered at TPJ [MNI -57, -49, 28] and L.Wernicke [MNI -65, -41, 9] (39–41). The coordinates in the CPC search space were *p_NZ_* ∈[0.52, 0.80] and *p_AL_* ∈[0.10, 0.26], as shown in **Supplementary Figure S2Bi**. The CPC positions (*p_NZ_*, *p_AL_*) remained the same across the subjects, and the distance between two adjacent CPC positions on the individual head model was approximately 2.83 mm.

#### 2.4.2 Calculate individual network targeting accuracy map

##### 2.4.2.1 TMS coil placements

We used SimNIBS 3.2 (42, 43) to segment T1 images of 134 participants and generate individual parameter spaces using their head surface nodes (44). For MDD, a total of 462 coil placements (462 positions × 1 orientation) were used for calculation for each individual. The coil orientation was fixed at 45° from the midline, and the coil handle pointed backward (45, 46) (**Supplementary Figure S2Aii**). For schizophrenia with AVH, the handle direction was perpendicular to the line between T3 and P3, which was around 23° from the midline measured within the Scalp Geometry-based Parameter (SGP) coordinate system (44). The handle is pointed backward (47) (**Supplementary Figure S2Bii**). A total of 246 coil placements (246 positions × 1 orientation) were set for each individual.

##### 2.4.2.2 Network targeting accuracy

We created an electric field for an individual coil placement using SimNIBS 3.2 and assigned default isotropic tissue conductivities (48). We selected the Magstim 70 mm figure-of-8 coil for electric field simulations (49), following which we created individual E-field weights using the previous pipeline (18). The practical scans for clinical uses range from 6 to 8 minutes (22, 50, 51), so we used half of right-to-left functional images of individuals (about 7 minutes) for the construction of individual FC. To determine individual FC, we masked the spatially normalized functional images of individuals in MNI space with a customized gray-matter mask consisting of 50740 voxels (18). We constructed a voxel-to-voxel correlation matrix (50740×50740) for each individual using Pearson’s correlation. Next, we built the TMS stimulation network of the coil placement using the individual E-field weights of the coil placement and individual voxel-to-voxel FC in MNI space. Finally, we determined the NTA by spatially anti-correlating the TMS stimulation network of the coil placement with the pathological networks derived from meta-analysis results for MDD or schizophrenia with AVH (18, 52, 53).

##### 2.4.2.3 Individual NTA map

We computed NTAs for all coil placements targeting the pathological network in MDD (**Supplementary Figures S2Ai, ii**) and in schizophrenia with AVH (**Supplementary Figures S2Bi, ii**). The resulting NTA maps were displayed on individual head surfaces for MDD (**Supplementary Figure S2Aiii**) and schizophrenia with AVH (**Supplementary Figure S2Biii**).

##### 2.4.2.4 Individual scalp site

We used the “Classic” method (18) to identify the optimal scalp position for stimulation, determined by the maximum value of the individual NTA map. The three-dimensional coordinates of the optimal scalp position served as the individual stimulation site.

### 2.5 Evaluate the instability of the personalized NTA model

We used three indices to evaluate the variance of the personalized NTA model, which included intrasession stimulation network similarity, intrasession NTA map similarity, and intraindividual distance.

Intrasession stimulation network similarity: To ensure the consistency of the stimulation network, it should be reliably determined by rsfMRI scans conducted on different days within the same individual. Therefore, the similarity between two separate stimulation networks obtained from the same individual should be maximized. The similarity was calculated as the correlation coefficient between the two stimulation networks.

Intrasession NTA map similarity: The NTA maps should be consistently determined by rsfMRI scans performed on different days within the same individual. The similarity of two separate NTA maps obtained from the same individual should be maximum. The similarity was also calculated as the correlation coefficient between the two NTA maps.

Intraindividual distance: Intraindividual distance was used as an evaluation index to determine the optimal scalp site consistently from rsfMRI scans conducted on different days within the same individual. The distance between the optimal scalp sites calculated from two separate rsfMRI scans from the same individual should be minimal and less than the ~1 cm spatial sensitivity of TMS (2), which is conventionally computed between two cortical sites (27, 29, 54) due to TMS’s spatial sensitivity being described on the cortex (2). First, we projected the individual scalp coordinate onto the cortex by finding the closest cortical site along the normal vector. Then, we transformed the cortical coordinate into MNI space using the subject2mni_coords function of the SimNIBS 3.2 package (43). Finally, the intraindividual distance was determined as the distance between the cortical coordinates of two separate scans conducted within the same individual.

### 2.6 Evaluate the feasibility of strategies for improving the stability of the personalized NTA model

Two strategies were implemented to enhance the stability of the personalized NTA model, namely the extension of rsfMRI scan durations and the cluster method. In this analysis, we present both strategies and the corresponding evaluation indexes.

i. Extend rsfMRI scan durations. We utilized longer rsfMRI scan durations. Specifically, we temporally concatenated two 14-minute 33-second runs per day to result in 28 minutes of data (27, 29). We then divided the 28 minutes of data into four different scan durations: 7 minutes, 14 minutes, 21 minutes, and 28 minutes. Similar to the 7-minute scan duration, we employed other scan durations in the personalized NTA model to compute intrasession stimulation network similarity, intrasession NTA map similarity, and intraindividual distance. This allowed us to observe the effect of longer rsfMRI scan durations on the stability of the personalized NTA model.

ii. “Cluster” method (29): We utilized alternative searching methods to identify optimal targets. Since the NTA model starts on the scalp surface, we modified the “cluster” method from Cash (29), which starts on the cortical surface. We identified contiguous clusters from the scalp-based individual NTA map and defined the center-of-gravity of the largest cluster as the target CPC position. This position’s three-dimensional coordinates were then used to determine the individual stimulation scalp site. To define clusters, we used the top x% of NTA values, with the threshold ranging from 0.5% to 70%. We also limited clusters to the supra-threshold CPC points using six neighborhoods on a 2-D plane. Apart from intraindividual distance, we also employed interindividual and ratio of interindividual-to-intraindividual distance to evaluate the feasibility of the personalized NTA model. The interindividual distance was used to ensure that the personalized targeting results did not converge to a fixed scalp site and that the optimal scalp site retained spatial diversity between individuals. Since individuals have different head sizes, the interindividual distance was measured in MNI space (55), which required projecting the individual scalp coordinate onto the cortex and transforming it into MNI space. The ratio of interindividual-to-intraindividual distance was used to ensure that the personalization methodologies maintained a smaller intraindividual distance and a larger interindividual distance, resulting in a higher ratio.

## 3 Results

### 3.1 Observe the variance of the personalized NTA model

**Figure 2:**
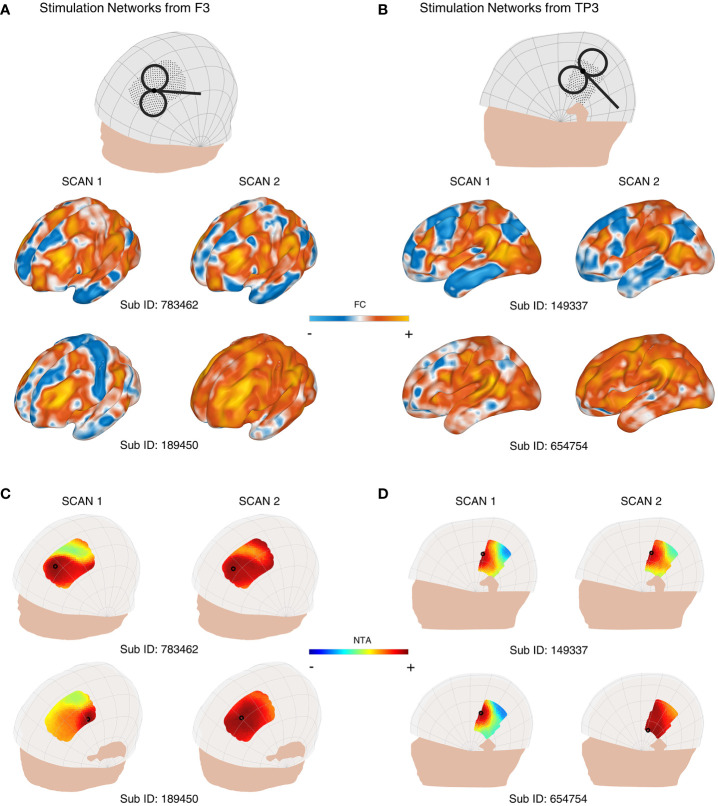
Variability of the personalized NTA model across different rsfMRI scans for targeting the pathological networks of MDD and schizophrenia with AVH. **(A)** shows the stimulation networks obtained from the F3 region, while **(B)** displays the stimulation networks from TP3 (the midpoint between T3 and P3). In the middle row of **(A)** (Sub ID 783462), the stimulation networks derived from two scans of the same target exhibit relatively high similarity. However, in the bottom row of **(A)** (Sub ID 189450), the same target produces different stimulation networks. NTA maps of representative individuals targeting the MDD pathological network are presented in **(C)**, and the schizophrenia with AVH pathological network is shown in **(D)**. The individual scalp sites (indicated by black circles) were determined using the classic method. The individuals depicted in the first columns of **(C)** show a wide range of target sites. In the middle row of **(C)** (Sub ID 783462), the target sites remain consistent across individuals on separate days. In contrast, in the bottom row of **(C)** (Sub ID 189450), the target sites vary among individuals on separate days. The optimal target site is marked with a black circle.

When we incorporated individual FC into the network targeting model, we observed significant variations in the stimulation network, NTA map, and the optimal target. For instance, **Figures 2A**, **2B** display the similarity and dissimilarity of the stimulation networks obtained from two 7-minute scans targeting points F3, and targeting the midpoint of T3 and P3 (TP3). As shown in the middle row of **Figure 2A** (i.e., Sub ID 783462), the stimulation network obtained from two scans of the same target was relatively similar. However, in the bottom row of **Figure 2A** (i.e., Sub ID 189450), two scans of the same target resulted in different stimulation networks. We computed the intrasession stimulation network similarity for F3 and TP3, and the correlation coefficients were 0.569 and 0.600, respectively (**Table 1**). For all coil placements in the search space in targeting MDD and schizophrenia with AVH pathological networks, the averaged intrasession stimulation network similarity was under 0.6.

**Table 1:** The variance of personalized NTA model.

	MDD	SZ with AVH
Intrasession Stimulation Network Similarity of F3 [r]	0.569 ± 0.012	N/A
Intrasession Stimulation Network Similarity of TP3 [r]	N/A	0.600 ± 0.011
Intrasession Stimulation Network Similarity [r]	0.553 ± 0.009	0.531 ± 0.011
Intrasession NTA map Similarity [r]	0.534 ± 0.031	0.778 ± 0.020
Intraindividual distance [mm]	31.265 ± 1.934	13.717 ± 1.129

N/A, not applicable.

Similarly, **Figures 2C, D** depict the individual NTA map and optimal target obtained from two 7-minute scans. As shown in the left column of **Figure 2C** and the left column of **Figure 2D**, the NTA map varied among individuals in the same scan, and optimal scalp sites were separated among individuals, which is consistent with previous studies (27, 29). In the top row of **Figure 2C** and the top row of **Figure 2D**, target sites were constant across individuals over separate days but variable among individuals across separate days in the bottom row of **Figure 2C** (i.e., Sub ID 189450), indicating the need for a stably personalized technique. The intrasession NTA map similarity for MDD and schizophrenia with AVH were 0.534 and 0.778, respectively.

When comparing individual target stability using 1 cm as the criterion, we found that the intraindividual distance was over 1 cm in both targeting the MDD pathological network and schizophrenia (SZ) with the AVH pathological network (**Table 1**). Additionally, we found that the stability was divided into two groups: the stable group (top row of **Figures 2C**, **2D**) and the unstable group (bottom row of **Figures 2C, D**). The statistics of the two groups showed that 102 people were in the unstable group for targeting the MDD pathological network, accounting for 76%; 32 people were in the stable group for targeting the MDD pathological network, accounting for 24%; 72 people were in the unstable group for targeting schizophrenia with AVH pathological network, accounting for 54%; 62 people were in the stable targeting schizophrenia with AVH pathological network, accounting for 46%.

### 3.2 Increase the stability of individual sites by extending rsfMRI scan duration

**Figure 3:**
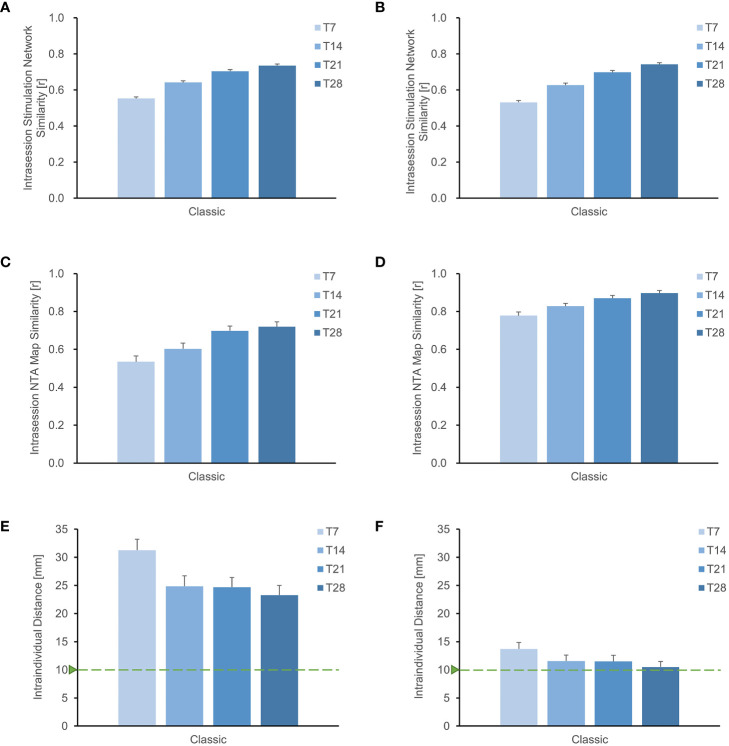
Improved stability of individual sites with longer rsfMRI scan duration. **(A)**, **(B)** depict the search space for MDD and schizophrenia with AVH, respectively, showing that the stability of the stimulation network gradually increases with longer scanning time. Similarly, for the entire search space of **(C)** MDD and **(D)** schizophrenia with AVH, the similarity of the NTA map also increases with extended scanning time. Additionally, when using the Classic method for optimizing the NTA map targeting either the **(E)** MDD network or the **(F)** schizophrenia with AVH network, it is evident that the intraindividual distance of the target decreases with longer scanning time. However, it remains higher than 1 cm.

We investigated the similarity of intrasession stimulation networks as scanning time increased. At a scanning time of 28 minutes, we observed that the intrasession stimulation network similarity at F3 was 0.750, while the intrasession stimulation network similarity at TP3 was 0.779 (**Supplementary Figure S3**). We also calculated the intrasession stimulation network for all points in the search space and found a consistent increasing pattern similar to that of a single target. In individuals targeting the MDD pathological network, the intrasession stimulation network increased from 0.553 to 0.740 as the scan duration increased from 7 to 28 minutes (**Figure 3A**). Similarly, in individuals targeting schizophrenia with an AVH pathological network, the intrasession stimulation network increased from 0.531 to 0.742 as the scan duration increased from 7 to 28 minutes (**Figure 3B**). These results indicate that retest reliability improves with a longer scanning time.

Furthermore, the extension of scanning duration resulted in improved similarity of intrasession NTA maps (**Figures 3C, D**). In individuals targeting the MDD pathological network, the correlation coefficient of the NTA map increased from 0.534 to 0.720 as the scan duration increased from 7 to 28 minutes. Similarly, in individuals targeting schizophrenia with AVH pathological network, the correlation coefficient of the NTA map increased from 0.778 to 0.897 with the same increase in scan duration. Both findings suggest that a longer scan time enhances the reliability of the NTA map.

Using the classic optimization method, we observed a gradual reduction in the distance of the optimal target within the individual with longer scanning time (**Figures 3E, F**). In individuals targeting MDD pathological network, the intraindividual distance reduced from 31.26 mm to 23.29 mm as the scan duration increased from 7 to 28 minutes. Similarly, in individuals targeting schizophrenia with AVH pathological network, the intraindividual distance reduced from 13.72 mm to 10.50 mm with the same increase in scan duration. However, both distances were still higher than 1cm. While longer scans decrease intraindividual distance, additional searching methods are required to improve target stability.

### 3.3 Increase the stability of individual sites with the cluster method

**Figure 4:**
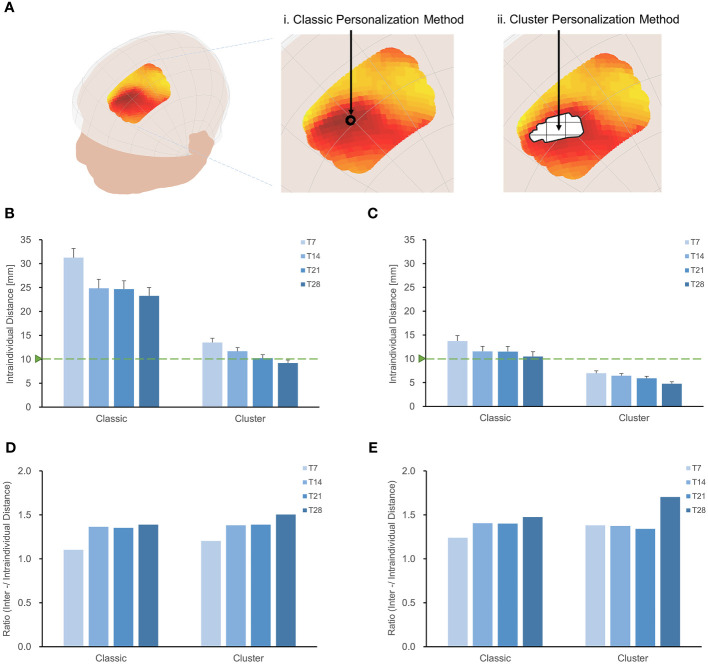
Increase the stability of individual sites with the cluster method. **(A)** Classic method and Cluster method. Intraindividual distances between personalized targets were displayed for different methodologies and scan durations (T7, T14, T21, T28), shown in targeting MDD pathological network **(B)** and schizophrenia with AVH pathological network **(C)**. Overall, the intraindividual distances with the classic method were greater than those achieved with the cluster method when targeting MDD and schizophrenia with AVH pathological networks. In both MDD and schizophrenia with AVH, the intraindividual distances using the cluster method and T28 were found to be smaller than the spatial sensitivity of TMS (2). Furthermore, the cluster method and T28 exhibited the highest interindividual-to-intraindividual distance ratios both in MDD **(D)** and schizophrenia with AVH **(E)**.

We evaluated the intraindividual distance of optimal sites (**Figure 4**) using the cluster method and 28-minute scan duration. The intraindividual distances were less than 1 cm in individuals targeting the MDD pathological network (9.23 ± 0.80 mm) and schizophrenia with AVH pathological network (4.76 ± 0.40 mm), as depicted in **Figures 4B, C**. The cluster method decreased intraindividual distances by 14 mm in MDD and 6 mm in schizophrenia with AVH, compared to the classic method and 28-minute scan duration. Although a longer scan duration results in a stable target, a shorter scan duration would be more practical. **Figure 4B** indicates that a 21-minute scan duration is the turning point for the stability of optimal targets.

We assessed the interindividual distance of optimal sites by employing the cluster method and a 28-minute scan duration (**Supplementary Figure S4**). In the case of targeting the MDD pathological network, the interindividual distance was 13.89 mm, while for targeting schizophrenia with the AVH pathological network, it was 8.11 mm. Utilizing the center-of-gravity calculation approach reduced intraindividual variance and interindividual variance, resulting in a 50% reduction in the interindividual distance compared to the classic method.

The cluster method proved to be more effective in identifying stable individual scalp sites for both targeting MDD and schizophrenia with AVH pathological networks when a 28-minute scan duration was employed, as demonstrated by higher interindividual-to-intraindividual distance ratios (**Figures 4C, D**). Furthermore, the ratios remained consistent even when the threshold was adjusted from 0.5% to 70% (**Supplementary Figure S5**). Specifically, the ratio was 1.51 for MDD and 1.70 for schizophrenia with AVH.

## 4 Discussion

After the three decades that rTMS has proved its utility in the treatment of psychiatric disorders, the substantial variance among individuals and disorders indicates the large room for technical improvement of the current TMS treatment (5, 56). Mounting evidence points to the association between treatment outcome and the targeted functional circuit in the brain (36, 57–60). Specifically, our previous finding that the functional specificity between the TMS stimulation network and the pathological network is associated with the treatment efficacy of MDD and schizophrenia AVH opens a way of guiding TMS using the functional MRI data. The NTA model holds the potential to guide personalized TMS treatments for psychiatric disorders (18). Reliable identification of the personalized optimal treatment site is an essential issue for addressing the feasibility of the personalized TMS network targeting and further validation of this approach (6, 27, 61).

In the current study, we implemented and evaluated the personalized network targeting model by incorporating the individualized fMRI. First, compared with the group-averaged FC, the individualized FC shows an advantage in retaining the inter-individual variance of the NTA hot spot [34.48 ± 0.21 mm] but at the cost of introducing intra-individual variance of [31.26 ± 1.93 mm]. Second, with integrating approaches enhancing the SNR, prolonging the scanning time, and spatial smoothing the NTA map, it is possible to substantially reduce the intra-individual variance to the level of [9.23± 0.63 mm] and relatively retain the inter-individual variance to the level of [13.89 ± 0.09 mm]. These results proved the feasibility of personalized network targeting of TMS.

In TMS-based treatment, the network architecture of the individual brain is a crucial factor in deciding the coil placement. When targeting associative cortical regions in treating psychiatric disorders, the anatomy-function association varies largely across individuals (62–64). Relatively, for a given pathological network associated with specific psychiatric disorders, the optimal stimulation site to target the pathological network varies from one individual to another, which may account for the individual difference in the treatment outcome (18). However, using group-averaged FC to guide TMS targeting may blur the functional heterogeneity of individual brains, and the one-site-fit-all solution has been questioned to be optimal for all individuals (6, 57, 65). As mounting evidence has demonstrated that individualized FC has advantages in depicting the variance of the engaged functional neuroanatomy (64, 66), personalized targeting based on individual FC could aid the identification of the optimal site for modulation the pathological network of psychiatric disorders.

However, it is crucial to acknowledge the variability observed in individual functional connectivity, which can arise from both desirable and undesirable variations. Desirable variations reflect significant inter-individual differences in network organization and connectivity strength (16, 28) in personalized TMS interventions. Conversely, undesirable variations stem from technical factors, measurement noise, or methodological limitations, which result in inaccurate estimation of functional connectivity and unreliable targeting sites (23, 27, 29, 31, 32). The application of the classic method in MDD with the personalized NTA model revealed an intraindividual distance exceeding 30 mm, highlighting the challenge of achieving personalization (27, 29, 30).

To address the variability arising from technical factors and establish a stable personalized NTA model, we employed two strategies: extending the scan duration for data acquisition and identifying optimal stimulation sites at the computational level. The duration of rsfMRI acquisition is critical in determining a stable optimal site (27, 29, 61). We assessed the intraindividual distance using four scan durations in MDD and schizophrenia with AVH. In the classic method, the 28-minute scan duration, we exhibited approximately 26% more reliability than the 7-minute scan duration in MDD, as measured by the intraindividual distance index, with a 23% improvement observed in schizophrenia with AVH. Considering that the optimal target may be an outlier singular point affecting the model’s stability, the cluster method utilized the similarity of NTA maps to identify reliable scalp sites by determining the center-of-gravity of the largest cluster rather than relying solely on peak values (29, 30). Our findings demonstrated that the intraindividual distance using the cluster method and a 28-minute scan duration was less than 1 cm, within the spatial sensitivity range of TMS (2), providing evidence of the effectiveness of the personalized NTA model. These strategies have partially addressed intra-individual variability.

Although capturing stable individual-specific functional network features may require several hours of scan data (16, 28), such an approach is impractical for therapeutic use. It is necessary to consider the tradeoff between scanning duration and the stability of individual targets (61). While a 28-minute scan is capable of acquiring a stable individual target, the turning point for stability was found to be a 21-minute scan, which is more practical. Additionally, alternative approaches for reliable functional connectivity estimation exist (67), including the use of a higher magnetic field to increase data quality (27, 68, 69) and multivariate estimates of functional connectivity (70). These strategies may be evaluated in future iterations of the personalized NTA model.

Furthermore, although strategies help decrease the variance of undesirable technical factors, minimizing intra-individual variability may lead to the loss of desirable variations, as indicated by a decrease in interindividual variations (**Supplementary Figure S4**). Preserving meaningful variations within individuals (16) and establishing a reasonable interindividual distance are crucial considerations. In an extreme example, when the interindividual distance is minimal, the best sites for all patients converge on one location. However, personalization becomes unnecessary if this one-site-fits-all method is successful. Clinical trials using neuronavigation targeting on a single site reported a response rate of approximately 50%, indicating that the optimal sites for all patients were dispersed (57). However, the criteria for the interindividual distance between these optimal sites remain uncertain. Using the personalized NTA model, we found that the interindividual distance in our analysis (13.89 mm) was close to the location variance of responders (19.45 mm), as demonstrated in **Supplementary Figure S6**.

Although we detected reasonable interindividual variance in our study, the feasibility of implementing a personalized NTA model also relies on whether the variance among individuals exceeds the variance induced by different fMRI scans. Consistent with the findings of a prior study (29), we observed that the interindividual variance outweighed the intraindividual variation in MDD and schizophrenia with AVH, as indicated by ratios greater than one. Furthermore, employing the cluster method alongside a 28-minute scan duration yielded higher ratios than other combinations. The ratio index demonstrates the ability of the model to balance desirable and undesirable variations.

It should be noted that our study has some limitations that need to be addressed through clinical experiments for validation. Firstly, it is important to recognize that the variance in TMS treatment may not solely be attributed to the variance in the stimulation network but also to the variance in the pathological network. In the current model, pathological networks are derived from coordinate-based meta-analysis, assuming the common network basis for a given disorder; however, recent progress in identifying bio-subtypes of disorders may provide a more accurate network target for TMS-based treatment (60, 71). Secondly, while a robust individualized NTA model set the path for accurate neuromodulation, it was insufficient to guide the accurate positioning of TMS coils in clinical trials. Retrospective validation is essential before applying the personalized NTA model. Thirdly, although investigating sex differences in targeting pathological networks falls beyond the scope of the current study, it necessitates further exploration to determine whether our proposed individualized network targeting model can elucidate sex differences in TMS treatment responses (72). For instance, in our dataset, we have observed variations in scalp-to-cortex distance (SCD) between sexes, as discussed in a prior article (72), leading to corresponding variances in the simulated E-field induced by the TMS coil (**Supplementary Figures S7**-**S10**). Given that our personalized NTA model considers individualized cranio-cortical correspondence, parallel to this study, our model holds the potential to unveil the biological mechanisms underlying sex differences in TMS treatments.

## 5 Conclusion

This study uses the HCP dataset to investigate the stability of a personalized connectivity-based network targeting model. Though incorporating individualized FC holds the potential to increase the precision of network targeting, we have demonstrated and quantified the instability that individualized FC can impose on the NTA model. We further demonstrated that incorporating two strategies previously used for reducing the FC instability, extending the rsfMRI scan duration and utilizing a spatial cluster method, can substantially reduce the intra-individual variance of the identified treatment site while retaining the inter-individual variance, suggesting its utility for guiding personalized TMS coil setting. Although retrospective validation is necessary, our current model offers a feasible approach to obtaining stable personalized TMS targets for the treatment of psychiatric disorders.

## Data availability statement

The original contributions presented in the study are included in the article/**Supplementary Material**. Further inquiries can be directed to the corresponding authors.

## Author contributions

ZC: Conceptualization, Data curation, Formal analysis, Investigation, Methodology, Software, Visualization, Writing – original draft. XX: Conceptualization, Investigation, Supervision, Writing – review & editing. CX: Software, Writing – review & editing. LW: Investigation, Writing – review & editing. YY: Conceptualization, Funding acquisition, Project administration, Supervision, Writing – review & editing. CZ: Conceptualization, Funding acquisition, Project administration, Supervision, Writing – review & editing.
