# Supplementary material for: Personalized connectivity-based network targeting model of transcranial magnetic stimulation for treatment of psychiatric disorders: computational feasibility and reproducibility
Source: Front Psychiatry. 2024 Feb 14;15:1341908. doi: 10.3389/fpsyt.2024.1341908 (PMC10899497; doi:10.3389/fpsyt.2024.1341908)
Supplement: Supplementary file 1 [file DataSheet_1.docx]

Supplementary Material

# Supplementary Results

**Figure S1. The comparison between optimal targets with different models under 28-minute scans.**

**Figure S2. Search space, coil orientation, and NTA map targeting MDD and schizophrenia with AVH pathological networks.**

**Figure S3. Similarity of stimulation network across scan times for a specific scalp location.**

**Figure S4. Interindividual distances.**

**Figure S5. The impact of cluster threshold on the results.**

**Figure S6. Comparison of interindividual distances among different models.**

**Figure S7. Comparison of scalp-to-cortex distances between males and females in the left dorsolateral prefrontal cortex.**

**Figure S8. Comparison of scalp-to-cortex distances between males and females in the left temporoparietal junction and the left Wernicke’s area.**

**Figure S9. Comparison of E-field and scalp-to-cortex distances between males and females in F3.**

**Figure S10. Comparison of E-field and scalp-to-cortex distances between males and females in TP3.**


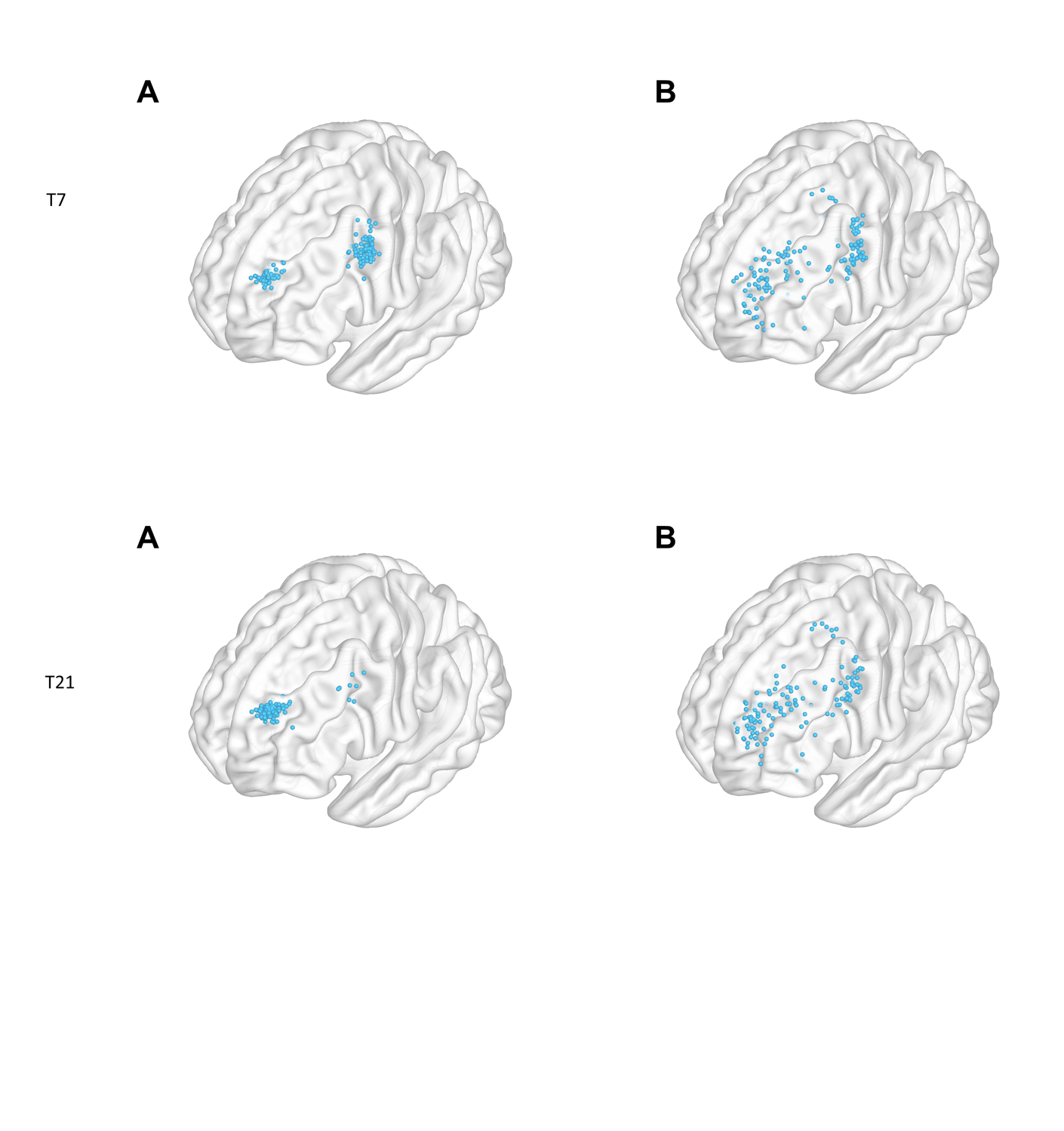


**Figure S1. The comparison between optimal targets with different models under 28-minute scans.** **(A)** Optimal targets with network targeting model using group-averaged FC. **(B)** Optimal targets with network targeting model using individual FC.


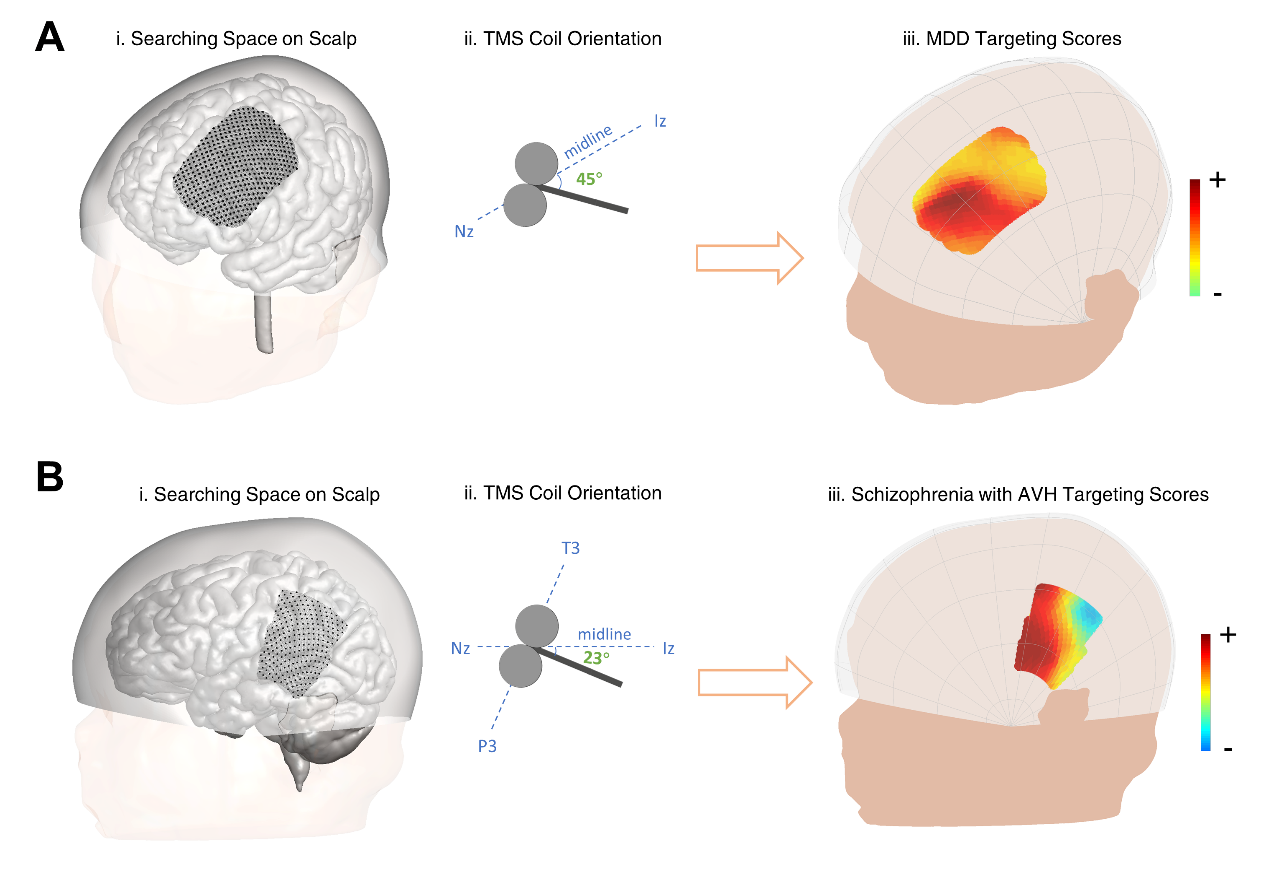


**Figure S2. Search space, coil orientation, and NTA map targeting MDD and schizophrenia with AVH pathological networks.** **(A)** Illustration of a representative individual's search space for MDD treatment (i). The search space is depicted by 462 positions (black dots). A fixed coil orientation (45° from the midline, ii) is maintained across the positions. NTA values are computed for each position-orientation pair, resulting in the corresponding MDD NTA map displayed within the search space (iii). **(B)** Illustration of a representative individual's search space for schizophrenia with AVH treatment (i). The search space is represented by 246 positions (black dots). A fixed coil orientation (23° from the midline, ii) is applied uniformly across the positions. NTA values are calculated for each position-orientation combination, leading to the corresponding schizophrenia with the AVH NTA map shown within the search space (iii).


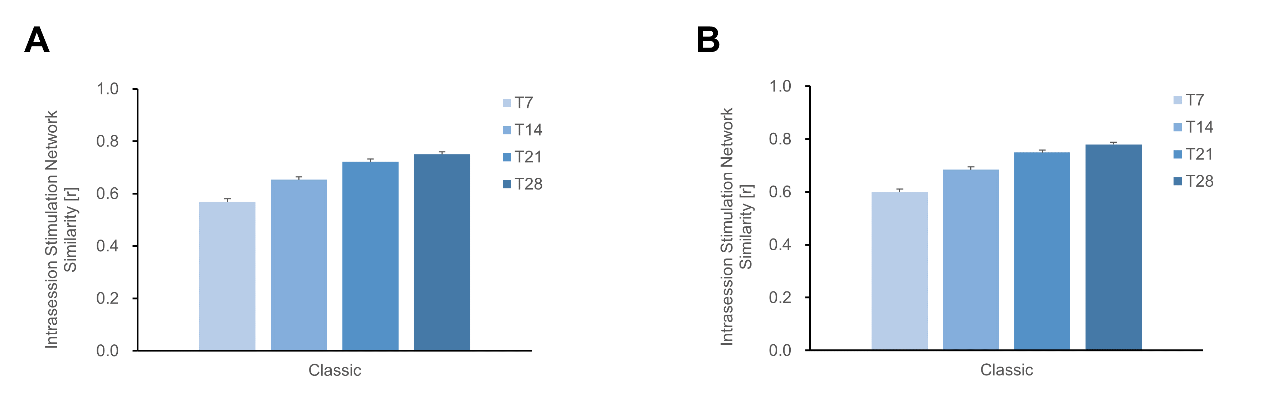


**Figure S3. Similarity of stimulation network across scan times for a specific scalp location**. The figure demonstrates the calculation of similarity between stimulation networks acquired during different scanning durations. Both **(A)** F3 and **(B)** midpoint of T3 and P3 (TP3) scalp locations show an increasing intrasession stimulation network similarity as the scanning time is extended.


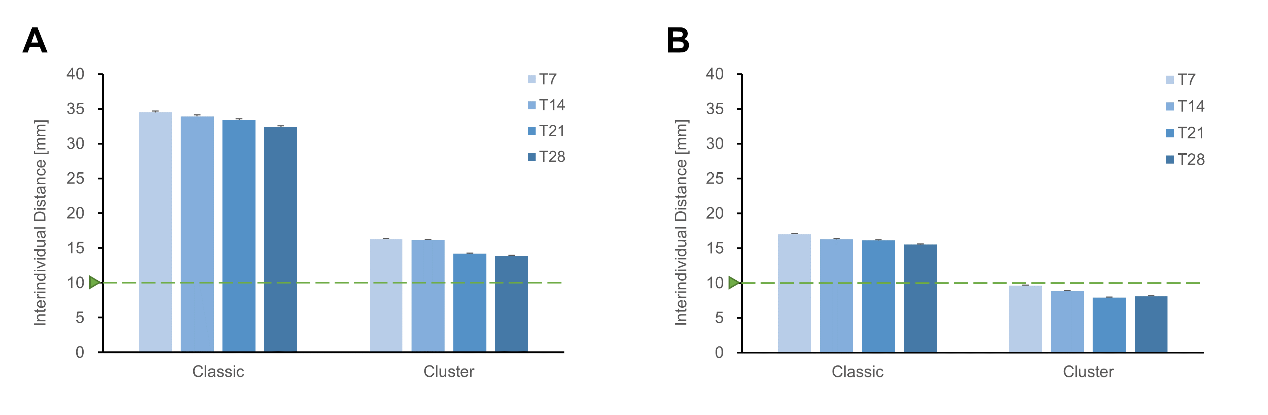


**Figure S4. Interindividual distances.** In order to assess the consistency across individuals, the distances between optimal targets obtained from the same scan but different individuals were calculated using the Classic or Cluster method. The results are presented for **(A)** targeting the MDD pathological network and **(B)** targeting schizophrenia with the AVH pathological network.


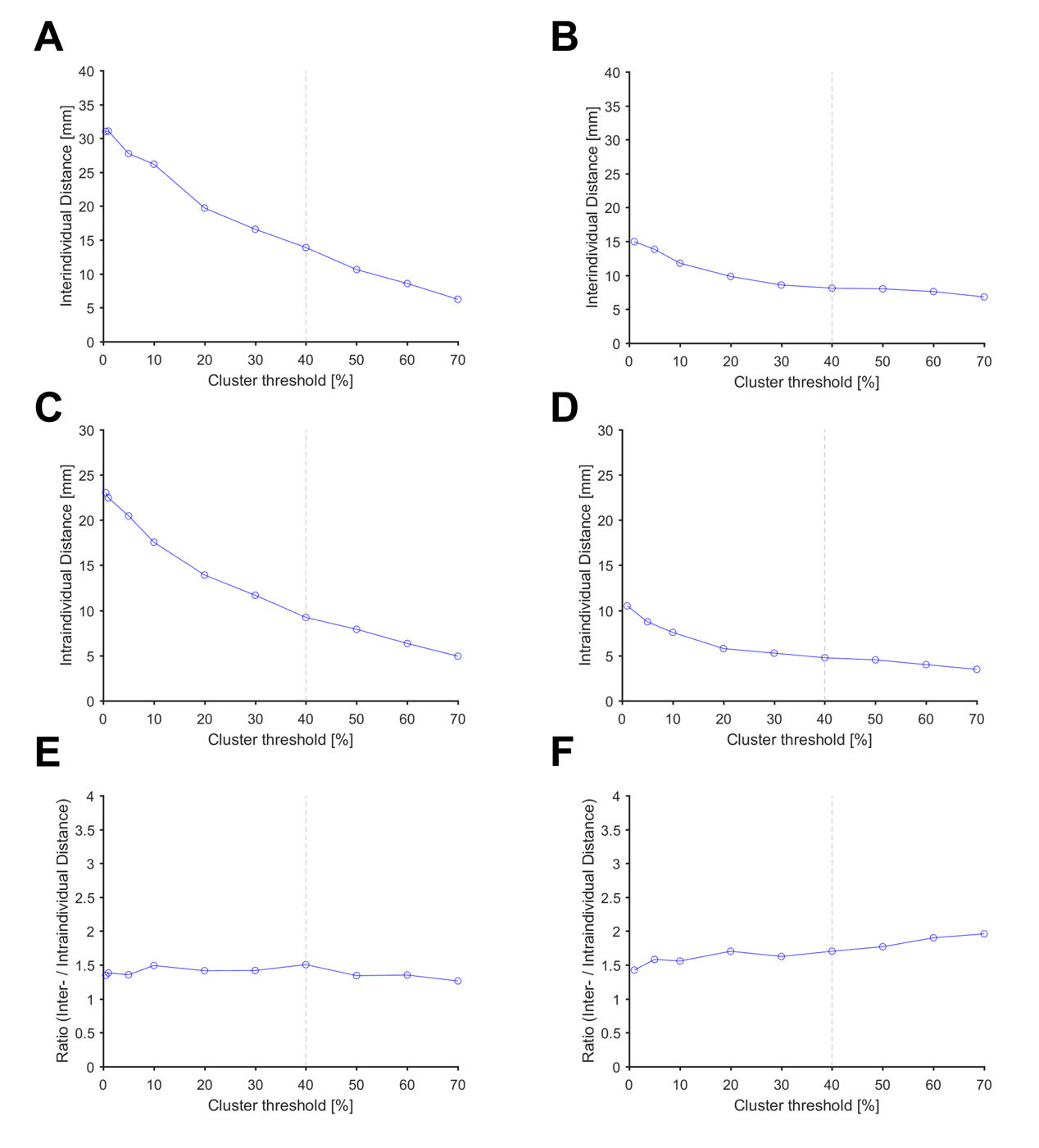


**Figure S5. The impact of cluster threshold on the results.** The interindividual distances were measured when targeting the MDD pathological network **(A)** and the schizophrenia with AVH pathological network **(B)**. The intraindividual distances were measured when targeting the MDD pathological network **(C)** and the schizophrenia with AVH pathological network **(D)**. The ratios were measured when targeting the MDD pathological network **(E)** and the schizophrenia with AVH pathological network **(F)**. To determine the appropriate threshold, the ratios were examined for both the MDD pathological network and the schizophrenia with AVH pathological network. It was observed that the ratios remained consistent for both networks. Therefore, a threshold of 40% was selected based on the maximum ratio value observed in the MDD network. Additionally, the interindividual distance obtained with the 40% threshold (13.89 mm) was comparable to the distance (14.11 mm) reported in a previous study (Cash et al., 2021).


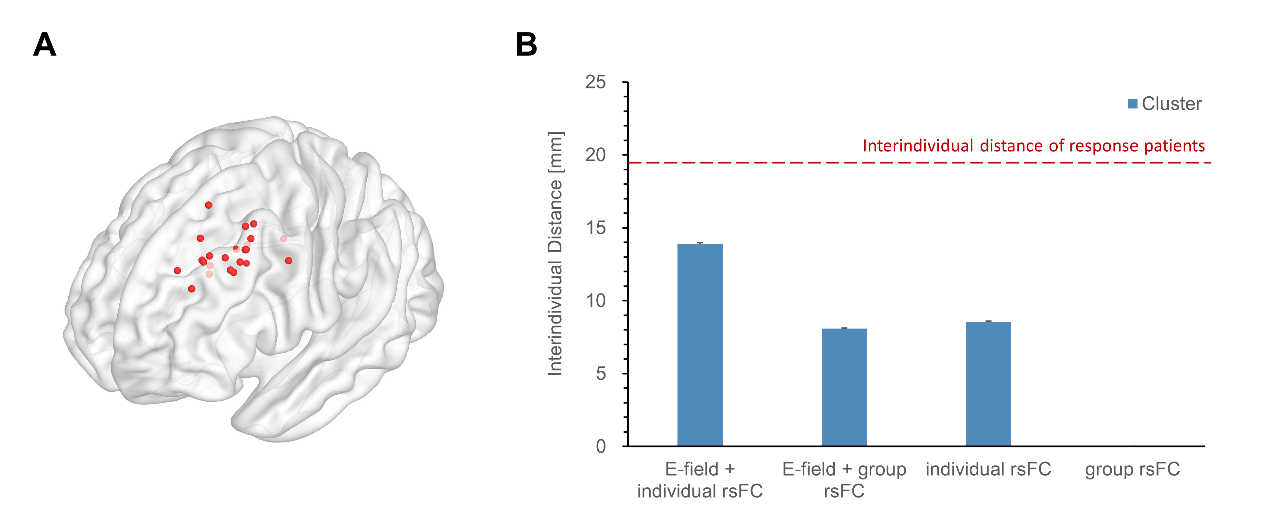


**Figure S6. Comparison of interindividual distances among different models.** TMS treatment responders, defined as individuals with a ≥50% reduction in clinical scores, were selected from previous studies (Paillère Martinot et al., 2010; Weigand et al., 2018). The cortical targeting sites are illustrated in **(A)**. The interindividual distance between these targeting sites of responders was used to measure the location variance among individuals. **(B)** Our findings revealed that the variance of individual targeting sites obtained using the personalized NTA model closely matched the location variance of the responders. Moreover, the variation observed when considering both individual E-field and individual FC was higher than when considering either individual E-field or individual FC alone. This suggests that personalized TMS treatment should account for both individual cortical characteristics and functional connectivity.


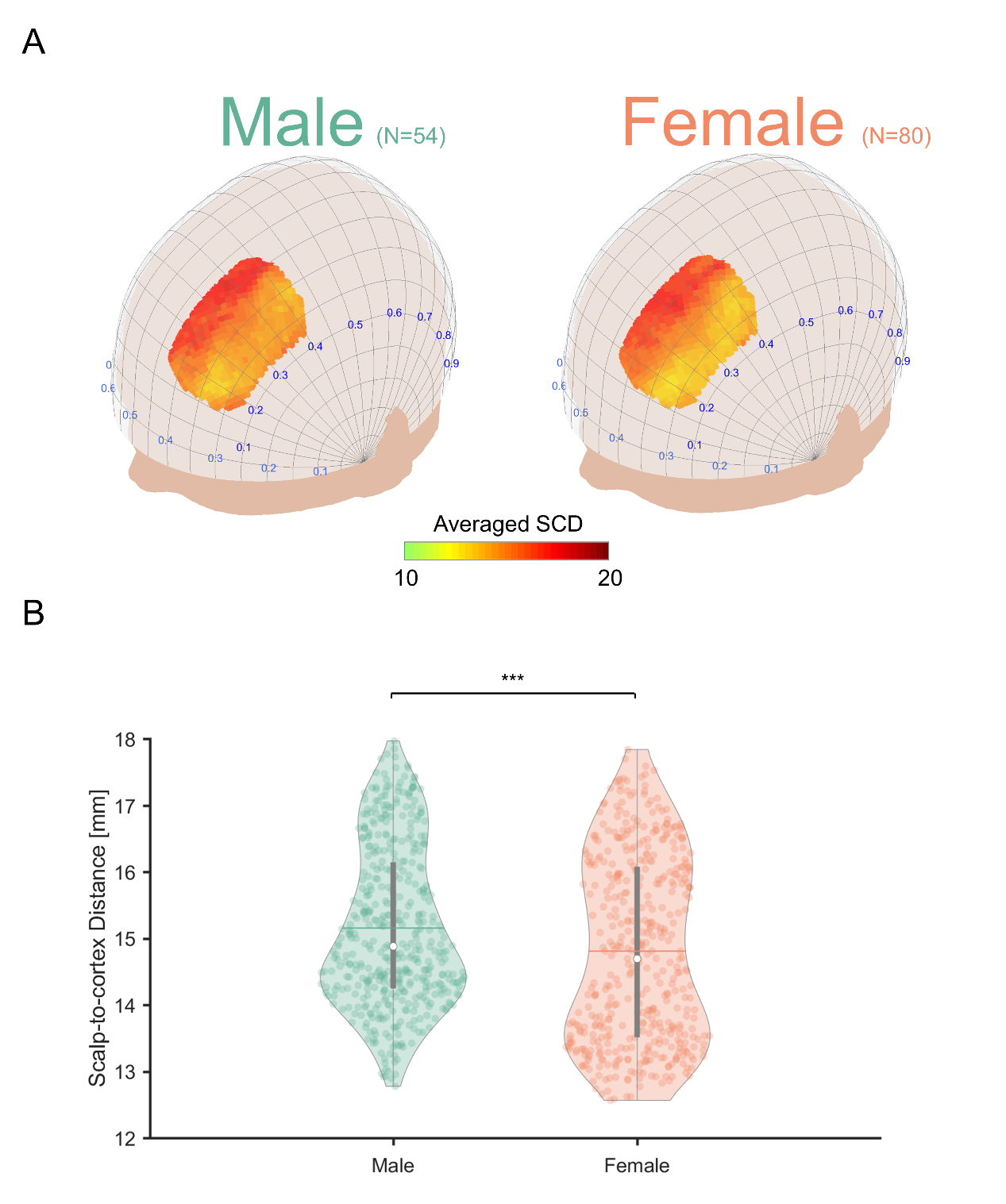


**Figure S7. Comparison of scalp-to-cortex distances between males and females in the left dorsolateral prefrontal cortex. (A)** Scalp-to-cortex distances (SCD) of males and females in the left dorsolateral prefrontal search space. Each CPC position represents an averaged SCD across males or females. **(B)** Sex difference of SCD. Each dot symbolizes an averaged SCD within a CPC position for males or females. The average SCD for males was significantly higher than that for females (Male: 15.15 ± 0.06 mm, Female: 14.81 ± 0.06 mm, t = 4.05, df = 922, *p* < 0.001).


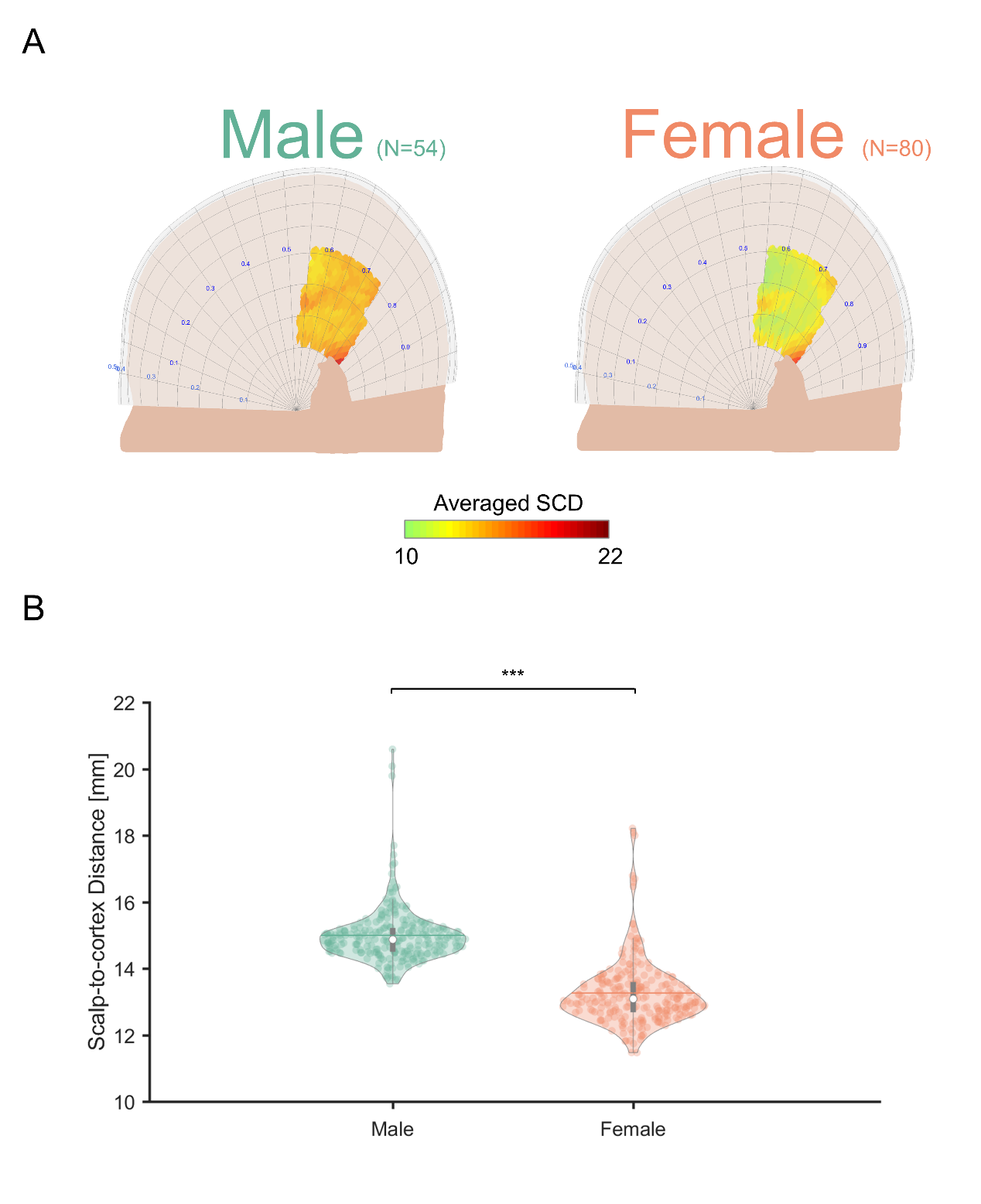


**Figure S8. Comparison of scalp-to-cortex distances between males and females in the left temporoparietal junction and the left Wernicke’s area. (A)** Scalp-to-cortex distances (SCD) of males and females in the search space of left temporoparietal junction and the left Wernicke’s area. Each CPC position represents an averaged SCD across males or females. **(B)** Sex difference of SCD. Each dot symbolizes an averaged SCD within a CPC position for males or females. The average SCD for males was significantly higher than that for females (Male: 15.00 ± 0.05 mm, Female: 13.26 ± 0.06 mm, t = 20.72, df = 490, *p* < 0.001).


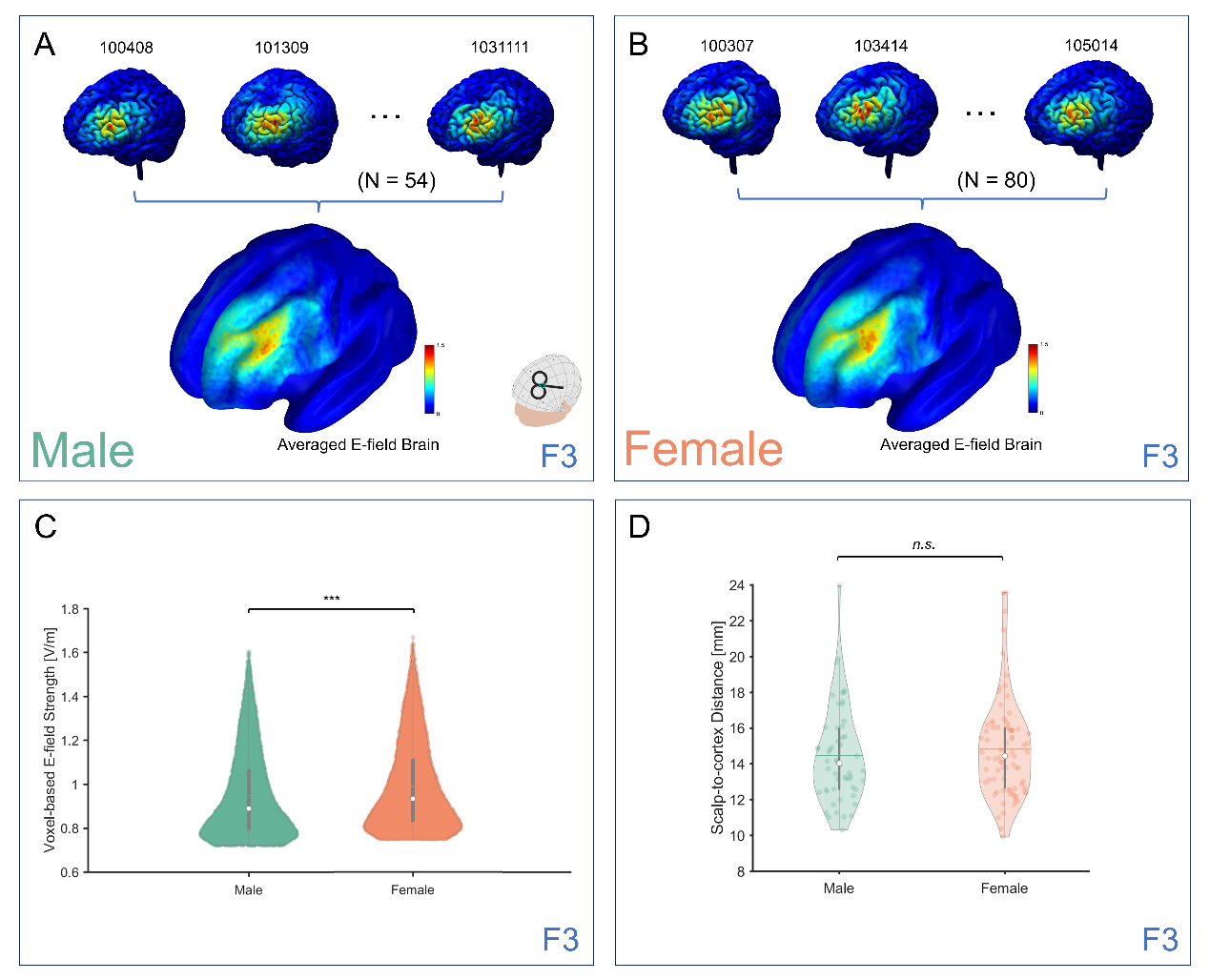


**Figure S9. Comparison of E-field and scalp-to-cortex distances between males and females in F3. (A)** The averaged E-field for males in F3. The TMS coil orientation depicted in Figure S2Aii. Individual’s E-field distributions in individual brains are displayed in the first row of the panel. We averaged normE outputs in MNI space across males, presented in the second row of the panel. **(B)** The averaged E-field for females in F3. **(C)** Sex difference of voxel-based E-field strengths in F3. The averaged E-field was extracted from gray matter voxels with E-field >90% of the maximum peak value (Makarov et al., 2021; Cao et al., 2023), defining the stimulated region by TMS. To mitigate the influence of outliers, the peak value was defined as the 99.9th percentile of the maximal E-field (Saturnino et al., 2019). Subsequently, voxel-based E-field strengths from the retained E-field map were compared between genders. The averaged E-field for females was found to be significantly higher than that for males (Male: 0.948 ± 0.002 V/m, Female: 0.988 ± 0.002 V/m, t = -14.769, df = 19892, *p* < 0.001). **(D)** Sex difference of SCD between sexes in F3. No significant differences were observed between the two groups (Male: 14.45 ± 0.36 mm, Female: 14.81 ± 0.31 mm, t = -0.76, df = 132, *p* = 0.45).


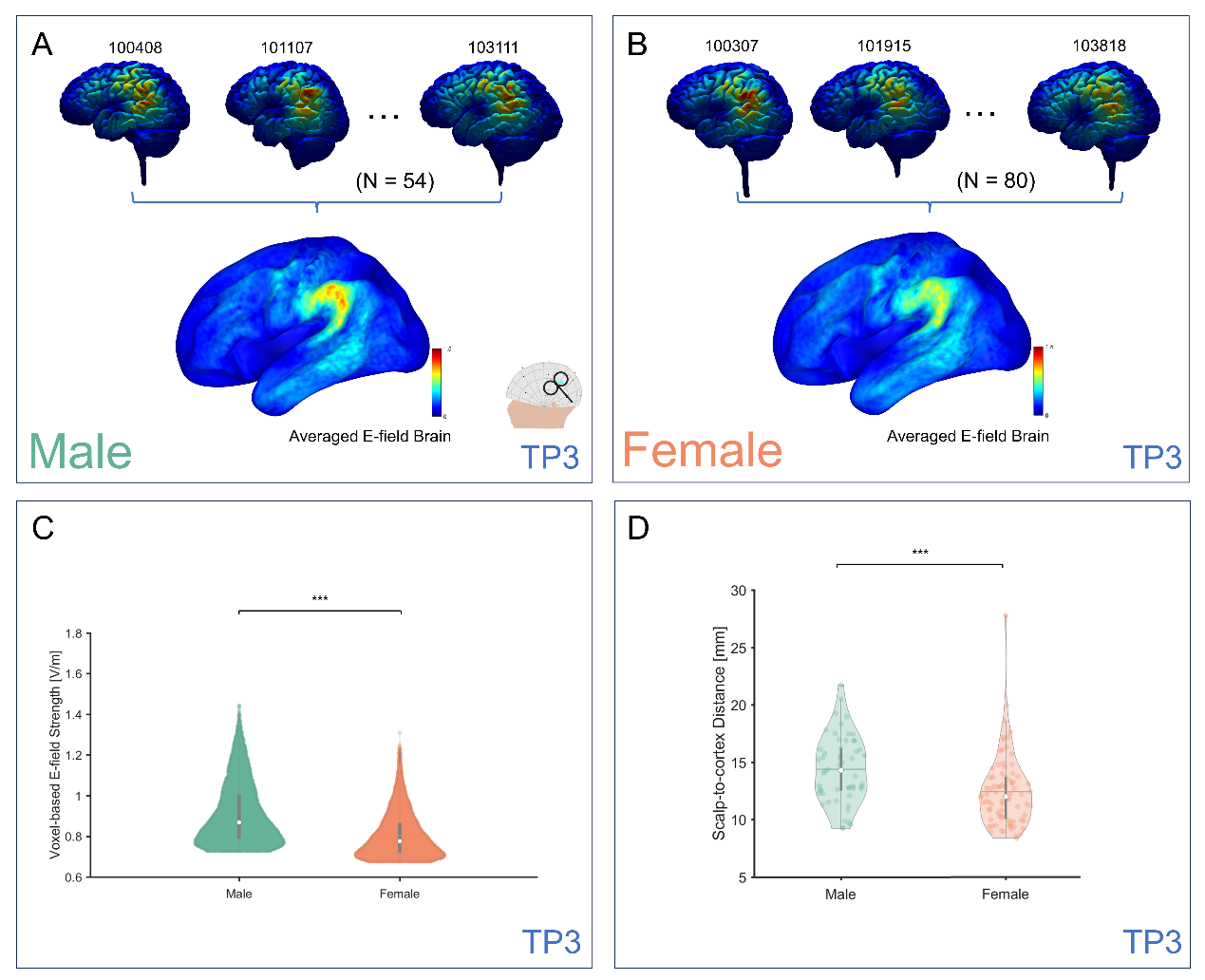


**Figure S10. Comparison of E-field and scalp-to-cortex distances between males and females in TP3. (A)** The averaged E-field for males in TP3. The TMS coil orientation depicted in Figure S2Bii. Individual E-field distributions in individual space are displayed in the first row of the panel. We averaged normE outputs in MNI space across males, presented in the second row of the panel. **(B)** The averaged E-field for females in TP3. **(C)** Sex difference of voxel-based E-field strengths in TP3. The averaged E-field was extracted from gray matter voxels with E-field >90% of the maximum peak value (Makarov et al., 2021; Cao et al., 2023), defining the stimulated region by TMS. To mitigate the influence of outliers, the peak value was defined as the 99.9th percentile of the maximal E-field (Saturnino et al., 2019). Subsequently, voxel-based E-field strengths from the retained E-field map were compared between genders. The averaged E-field for males was found to be significantly higher than that for females (Male: 0.910 ± 0.001 V/m, Female: 0.804 ± 0.001 V/m, t = 62.808, df = 22783, *p* < 0.001). **(D)** Sex difference of SCD in TP3. The average SCD for males was significantly higher than that for females (Male: 14.39 ± 0.40 mm, Female: 12.41 ± 0.34 mm, t = 3.73, df = 132, *p* < 0.001).

## References

Cao, Z., Xiao, X., Zhao, Y., Jiang, Y., Xie, C., Paillère-Martinot, M. L., et al. (2023). Targeting the pathological network: Feasibility of network-based optimization of transcranial magnetic stimulation coil placement for treatment of psychiatric disorders. *Front. Neurosci.* 16, 1–15. doi: 10.3389/fnins.2022.1079078.

Cash, R. F. H., Cocchi, L., Lv, J., Wu, Y., Fitzgerald, P. B., and Zalesky, A. (2021). Personalized connectivity-guided DLPFC-TMS for depression: Advancing computational feasibility, precision and reproducibility. *Hum. Brain Mapp.* 42, 4155–4172. doi: 10.1002/hbm.25330.

Makarov, S. N., Wartman, W. A., Noetscher, G. M., Fujimoto, K., Zaidi, T., Burnham, E. H., et al. (2021). Degree of improving TMS focality through a geometrically stable solution of an inverse TMS problem. *Neuroimage* 241. doi: 10.1016/j.neuroimage.2021.118437.

Paillère Martinot, M. L., Galinowski, A., Ringuenet, D., Gallarda, T., Lefaucheur, J. P., Bellivier, F., et al. (2010). Influence of prefrontal target region on the efficacy of repetitive transcranial magnetic stimulation in patients with medication-resistant depression: A [18F]-fluorodeoxyglucose PET and MRI study. *Int. J. Neuropsychopharmacol.* 13, 45–59. doi: 10.1017/S146114570900008X.

Saturnino, G. B., Puonti, O., Nielsen, J. D., Antonenko, D., Madsen, K. H., and Thielscher, A. (2019). SimNIBS 2.1: A Comprehensive Pipeline for Individualized Electric Field Modelling for Transcranial Brain Stimulation. *Brain Hum. Body Model.*, 3–25. doi: 10.1007/978-3-030-21293-3_1.

Weigand, A., Horn, A., Caballero, R., Cooke, D., Stern, A. P., Taylor, S. F., et al. (2018). Prospective Validation That Subgenual Connectivity Predicts Antidepressant Efficacy of Transcranial Magnetic Stimulation Sites. *Biol. Psychiatry* 84, 28–37. doi: 10.1016/j.biopsych.2017.10.028.
